# Using Genetics to Evaluate the Success of a Feral Cat (*Felis catus*) Control Program in North-Western Australia

**DOI:** 10.3390/ani9121050

**Published:** 2019-12-01

**Authors:** Saul Cowen, Lucy Clausen, Dave Algar, Sarah Comer

**Affiliations:** 1 Biodiversity and Conservation Science, Department of Biodiversity, Conservation and Attractions, P.O. Box 51, Wanneroo, WA 6946, Australia; dave.algar@dbca.wa.gov.au; 2 Parks and Wildlife Service, Department of Biodiversity, Conservation and Attractions, 20 Nimitz Street, Exmouth, WA 6707, Australia; lucy.clausen@dbca.wa.gov.au; 3 Parks and Wildlife Service, Department of Biodiversity, Conservation and Attractions, 120 Albany Highway, Albany, WA 6330, Australia; sarah.comer@dbca.wa.gov.au

**Keywords:** invasive species, genetic diversity, population dynamics, cats

## Abstract

**Simple Summary:**

The management of invasive species is a major challenge for the conservation of biodiversity globally. One technique that has been widely used to control feral cats (*Felis catus*) and red foxes (*Vulpes vulpes*) in Western Australia is the aerial broadcast of toxic baits, but assessing its efficacy can be difficult. Here, we report on a method of evaluating the effectiveness of this method for the abatement of feral cats using genetic analysis techniques. However, our results were unable to provide robust evidence that, over a five-year program, baiting had a detrimental impact on both genetics and demography in this population, and the results were not significant. Monitoring the impact of control programs in this way may provide valuable information to managers on the effectiveness of their management strategy, but further refinement of the methodology is recommended.

**Abstract:**

The feral cat has been implicated in the decline and extinction of many species worldwide and a range of strategies have been devised for its control. A five-year control program using the aerial broadcast of toxic Eradicat^®^ baits was undertaken at Fortescue Marsh in the Pilbara region of north-western Australia, for the protection of biodiversity in this important wetland area. This program has been shown to have had a significant detrimental effect on cats in this landscape, but the long-term impact is difficult to ascertain. We assessed population genetics across three cohorts of feral cats sampled as part of the control program. We also compared cat populations in natural habitats and around human infrastructure. A key challenge in any study of wild animal populations is small sample sizes and feral cats are particularly difficult to capture and sample. The results of this study superficially appear to suggest promising trends but were limited by sample size and many were not statistically significant. We find that the use of genetic techniques to monitor the impact of invasive species control programs is potentially useful, but ensuring adequate sample sizes over a long enough time-frame will be critical to the success of such studies.

## 1. Introduction

Invasive species are listed as one of the key threatening processes for biodiversity globally [1] and have been implicated in the decline and extinction of a number of native species [2,3]. The domestic (or ‘feral’) cat (*Felis catus*) is listed as one of the world’s worst invasive species [4] and their detrimental impact on Australian native species is widely recognised [5,6]. Among the control strategies for cats is the use of toxic baits, which can be applied at a landscape-level by aerial broadcast from an aircraft. This method has been implemented by the Western Australian Department of Biodiversity, Conservation and Attractions (DBCA) and has been shown to have had a significant impact on red foxes (*Vulpes vulpes*) in reserves managed by the department across the state [7]. Aerial broadcast baiting has also been used to control feral cats using Eradicat^®^ baits in several locations in Western Australia [8]. In these cases, effectiveness of the baiting program has been assessed by (a) responses in populations of native species or (b) mortalities of individual animals, fitted with telemetry collars prior to baiting or (c) occupancy models using detections on remote cameras [9]. For example, a five-year control program at Fortescue Marsh in the Pilbara region of Western Australia used (b) and (c) to show that a landscape-scale Eradicat^®^ baiting program had a significant detrimental impact on cats on an annual basis [9]. Monitoring of the longer-term impact of baiting on invasive predator populations is much harder to quantify, especially in a location such as Fortescue Marsh, where there are few barriers to reinvasion from outside the area that is subject to baiting. Furthermore, methods that require the assumption of a closed system (such as occupancy modelling) are not appropriate when the dataset extends beyond the baiting period in a given season.

Genetic diversity in vertebrate populations is understood to be an important factor in extinction risk [10,11]. Populations with relatively low genetic diversity are often less viable than those with higher variability [12,13,14,15,16]. The diversity of genes within a group of loci can be used to infer quantitative genetic measures [17] including inbreeding, relatedness, population structure and effective population size (Ne) [18]. Ne, in the context of a census population, is the number of reproductive individuals contributing offspring to that population [19]. In turn, these estimates have been used to evaluate the impact of a control strategy on a population of an invasive species [20,21,22], including by inferring reductions in allelic diversity, heterozygosity and Ne as indicative of a reduction in the number of reproductive individuals in a system [23]. Spatial patterns of gene flow and relatedness may also contribute to our knowledge of dispersal and other behaviours, which may help to inform future augmentation of control measures [23]. For example, sex-biased dispersal may result in a different impact of a broad-scale control program on males and females.

To evaluate whether the success of the Fortescue Marsh control program on an annual basis resulted in a measurable inter-annual impact on population genetics, 11 polymorphic microsatellites were used to genotype a suite of feral cats captured as part of this program [9,24]. The primary objective of these captures was to fit telemetry collars to assess bait-uptake and landscape use. As such, no animals were captured in non-baited areas, so no experimental control was imposed. Genetic data from captured cats were used to conduct population-wide genetic analyses: Hardy-Weinberg Equilibrium (HWE) (a deviation from may be indicative of a number of factors e.g., genotyping errors, inbreeding, non-random mating), Ne and spatial autocorrelation of relatedness across the landscape. In addition, levels of inbreeding, estimates of migration rate and geographic population structure were calculated between ‘marsh’ cats and ‘camps’ cats that were captured in the vicinity of mining infrastructure, which currently fringes the northern edge of the Marsh. Importantly, cats in the ‘camps’ population were unlikely to be subject to baiting, since areas of mining infrastructure were excluded from the bait treatment area. However, the ‘camps’ may potentially be a source of immigrants into the ‘marsh’ population, thereby undermining ongoing efforts on the marsh itself. These groups were treated arbitrarily as separate populations, but the veracity of this assumption was tested.

## 2. Materials and Methods

**Figure 1:**
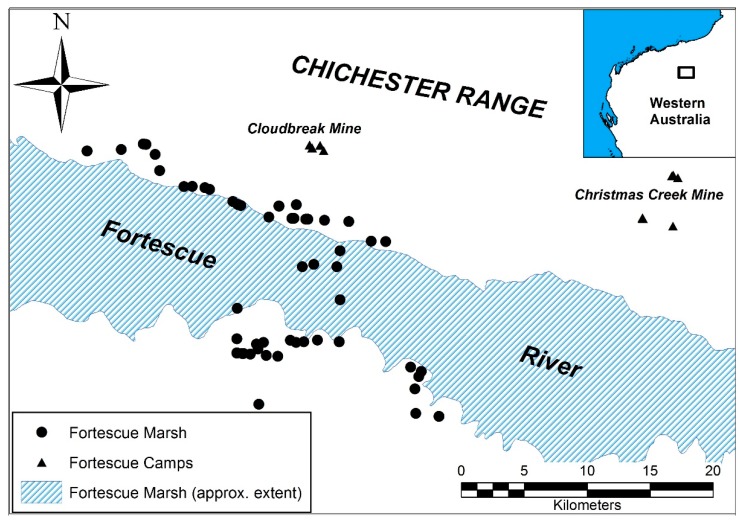
Sampling locations of feral cats for genetic analysis between 2012 and 2016.

Fortescue Marsh is a large wetland that is subject to periodic inundations as large as 1000 km^2^ in the last 20 years, with an overall catchment size of 1300 km^2^ [25]. The Marsh is situated in the Hamersley Basin in the Pilbara region of north-west Australia (Figure 1) and has a semi-arid climate (bordering on arid), with a mean annual rainfall of 312 mm for nearby Newman [26]. Inundation events are linked to extreme rainfall events caused by tropical cyclones [25]. Vegetation falls into 21 separate communities (Markey unpub. data), ranging from woodland dominated by *Eucalyptus* spp. and *Acacia aneura* at the margins of the Marsh to spinifex (*Triodia* spp.) grassland to halophytic chenopod shrubland in the centre of the Marsh [24,26]. Fortescue Marsh is a wetland of national significance and is listed under the Directory of Important Wetlands in Australia (reference no. WA066; [27]). Several species of national conservation significance have been recorded on the Marsh, including night parrot (*Pezoporus occidentalis*) and greater bilby (*Macrotis lagotis*) [28,29] as well a number of migratory bird species listed under the Bonn Convention [24,30,31]. Many of these species could potentially benefit from the control of feral cats in this area.

Fortescue Marsh is adjacent to the Cloudbreak and Christmas Creek iron ore mining areas, which are operated by Fortescue Metals Group Limited (FMG). On behalf of FMG, DBCA implemented the Fortescue Marsh Baiting Plan [32] to satisfy Condition 16 of the EPBC Act approval 2010/5706, which is aimed at improving protection and long-term conservation of EPBC Act listed species in the Fortescue Marsh [24]. The program involved the aerial broadcast of Eradicat^®^ baits (Department of Biodiversity, Conservation and Attractions, Perth, Australia) once a year for five years (2012–2016) and monitoring of both feral cats and native fauna was to be conducted to assess the efficacy of the program. Eradicat^®^ baits contain 4.5 mg directly-injected sodium monofluoroacetate (compound 1080) and were broadcast at the approved prescription of 50 baits per km^2^. Flight transects 1 km apart aimed to achieve a spread per bait drop of 200 × 40 m. The baiting program was conducted in winter when conditions were cool and dry and when live prey was understood to be least abundant [9]. Low humidity and precipitation were also important to maximise the persistence of the toxin in the baits. The shape of the baited area changed from year to year to accommodate varying levels of inundation on the marsh itself, with approximately 838 km^2^ baited in 2012 and 2013 and approximately 920 km^2^ in 2014–2016.

One technique for monitoring the impact of baiting on feral cats involved the capture and fitting of GPS telemetry collars to individual feral cats in the baiting area to monitor behaviour (e.g., spatio-temporal movement; home-ranges; rate of detection on remote cameras) and ultimately the uptake of baits [9,24]. Individuals captured for collaring had tissue samples taken for the purposes of DNA analysis. Cats were captured using padded leg- hold traps Victor ‘Soft Catch’ ^®^ (Woodstream Corp., Lititz, PA, USA) using a combination of cat urine and faeces as the attractant. Captures took place between 2012 and 2016, during late autumn-early winter at least three weeks prior to baiting, with a total of 65 individuals being sampled from the Marsh area (‘marsh’ cats) (Figure 1). In addition, 14 samples were collected by project staff and FMG personnel who conducted the trapping of cats around infrastructure at both Cloudbreak and Christmas Creek mine-sites (‘camps’ cats) (Figure 1) using conventional cage-traps and on an ad-hoc basis, not necessarily simultaneously with ‘marsh’ cat captures. While these samples were ancillary to the study of the ‘marsh’ population, they were included with the aim of understanding whether there were any observable genetic differences between the ‘camp’ cohort and the ‘marsh’ cats and to provide an estimate the rate of migration between the two populations (a potentially important consideration for future management). Cats were sampled across a total area of approximately 800 km^2^ and the ratio of males to females was 41:38. The sample array for the ‘marsh’ cats was further divided into three temporal cohorts of comparable size: 2012–2014 (n = 26); 2015 (n = 20); 2016 (n = 19). While these sample sizes were relatively small, they were comparable to those used in a previous study [23] which identified changes in patterns of genetic diversity, albeit in a very different ecological system (Hawai’i Island, Hawai’i).

Extraction of DNA from tissue samples and subsequent analysis was conducted by Y. Hitchen (Helix Molecular Solutions). Genotypes were generated using 11 polymorphic short-tandem repeat (microsatellite) markers characterised for felids [33,34] (Table 1) and were visualised in a MS Excel spreadsheet.

Genetic diversity was quantified using mean alleles per locus (AL) across all 11 microsatellites, as well as mean allelic richness (AR) between spatial and temporal cohorts and these were calculated using HP-RARE [35] using the smaller of the sample cohorts, ‘camps’ (n = 14) for AR. This package was also used to construct allele rarefaction curves (this time using the total sample sizes for both populations (n ‘marsh’ = 65; n ‘camps’ = 14)) to predict whether the given samples sizes were sufficient to obtain the majority of the available genetic diversity in this population.

GENEPOP (version 4.2, Université Montpellier 2, Montpellier, France) [36] was used to calculate summaries of departures from HWE, from which estimates for inbreeding (FIS) and pairwise fixation index (FST) were derived as well as migration rate (Nm) (private allele method [37] between ‘marsh’ and ‘camps’ cats. Significance levels for FIS and FST were adjusted for multiple comparisons using a Bonferroni correction. Nm was also calculated from FST estimates using the formula [38]:

Nm = (1/FST − 1)/4.

Estimates of Ne were calculated for ‘marsh’ and ‘camps’ cohorts, as well as across the three temporal cohorts, in NeESTIMATOR (version 2.01, University of Queensland, St Lucia, Australia) [39] using the Linkage Disequilibrium (LD) and Molecular Coancestry (MC) methods. Predictions of geographic genetic structure were made in STRUCTURE (version 2.3.4, Stanford University, Stanford, CA, USA) [40] using a Bayesian model computation. POPULATIONS (version 1.2.31, Centre National de la Recherche Scientifique, Gif-sur-Yvette, France) [41] was used to estimate genetic distance between all individuals in the dataset by constructing a Neighbour-Joining tree (using Nei’s minimum genetic distance model [42]) which was visualised in MEGA (version 6.06, Arizona State University, Tempe, AZ, USA) [43].

To assess how genetic distance correlates with geographic distance across the landscape, predictions of spatial autocorrelation were made using GENALEX (version 6.502, The Australian National University, Acton, Australia) [44,45] using the Spatial > Single Population Module with 999 permutations and 1000 bootstraps. Spatial autocorrelation results were produced for all individuals on the Marsh, as well as for separate male and female cohorts, to investigate whether sex-biased dispersal can be inferred to occur on the Marsh.

## 3. Results

### 3.1. Genetic Diversity and F-Statistics

**Figure 2:**
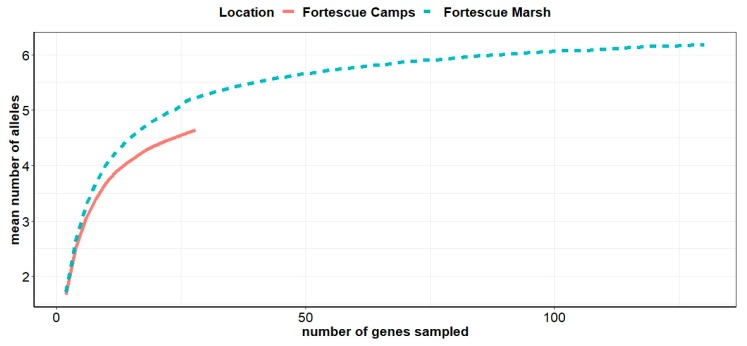
Allele rarefaction curve for samples of ‘marsh’ and ‘camps’ populations, indicating the proportion of the available diversity that has been sampled for the given set of markers.

Allele rarefaction curves were constructed for both populations (Figure 2) and indicated that, while the curve for the ‘marsh’ population reaches an asymptote (and consequently this population has had most of its genetic variation sampled), the curve for the ‘camps’ population was far from asymptotic and therefore the sample size of 14 may be insufficient to capture the majority of available genetic variation.

Allelic diversity was considerably higher in the ‘marsh’ population rather than the ‘camps’, with a value for AL of 6.18 (±SD 2.31) for the ‘marsh’, compared to 4.64 (±SD 1.50) for the ‘camps’ population (Table 2). Accounting for differential sample sizes between putative populations after rarefaction with a sample size of 14, AR was 5.16 (±SD 1.39) for the ‘marsh’ population (c.f. 4.64 for ‘camps’).

**Figure 3:**
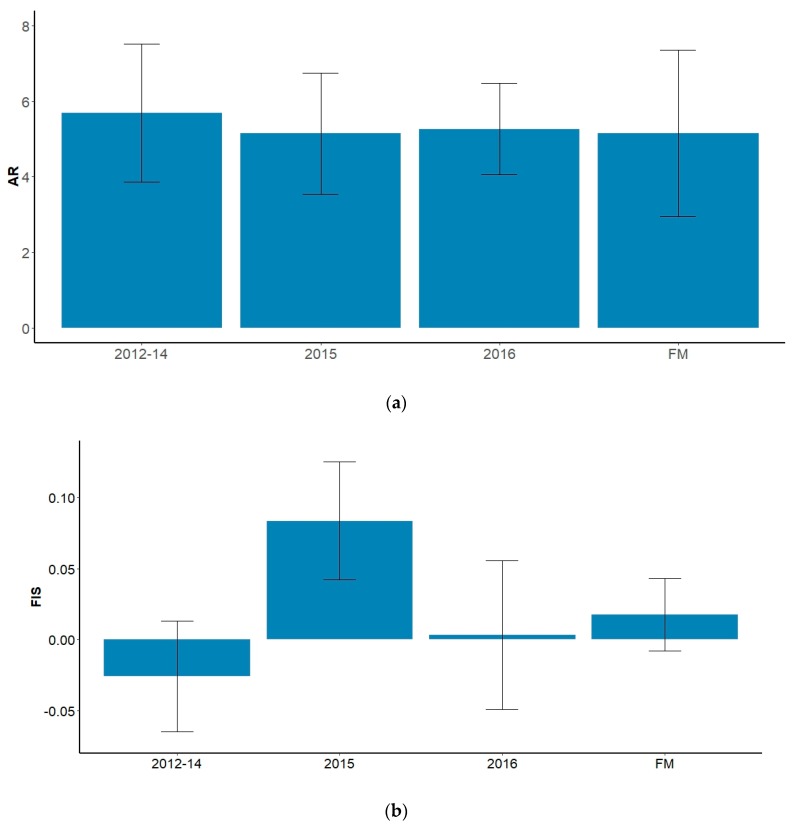
(**a**) Estimates of mean allelic richness (AR) and (**b**) inbreeding coefficient (FIS) for three temporal cohorts of feral cats from the ‘marsh’ population at the Fortescue Marsh study site, as well as for the overall population (FM), included for context. Error bars for AR indicate standard deviation across all loci; error bars for FIS are standard errors.

Low allelic diversity also corresponded to lower observed heterozygosity in the ‘camps’ population with a FIS value of 0.036 compared to 0.018 for the ‘marsh’ sample (Table 2). The level of inbreeding in the ‘camps’ population was statistically significant at the *p* < 0.05 level but not after a Bonferroni correction was applied. FST values were generally low across all loci and a mean value of 0.012 indicates that just 12% of observed variance was down to population subdivision (Table 2). Across cohorts, mean alleles per locus and mean allelic richness showed minor fluctuations that were not statistically significant (Figure 3). There was an apparent increase in FIS values from −0.0258 in 2012–2014 to 0.0837 in 2015, declining to 0.0034 in 2016 (Figure 3), but again the overall trend was not significant.

### 3.2. Effective Population Size

**Figure 4:**
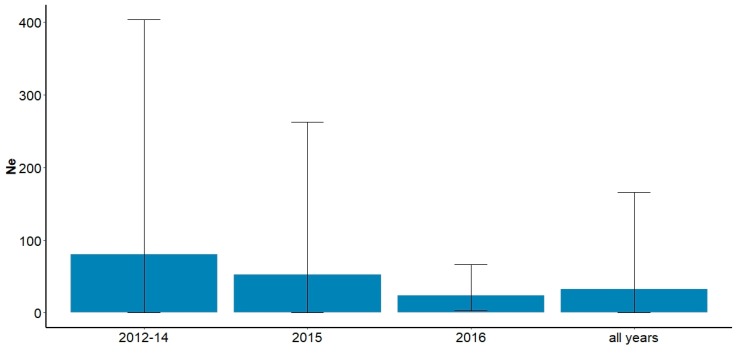
Estimates of effective population size (Ne) across three cohorts of feral cats from Fortescue ‘marsh’ population (n: 2012–2014 = 26; 2015 = 20; 2016 = 19) using the Molecular Coancestry (MC) method in NeEstimator (version 2.01) [40]. Error bars reflect calculated 95% confidence intervals (CI). Values of Ne and CI that exceed chart area are infinite (∞).

Overall Ne values across all years for Fortescue ’marsh’ cats were calculated as 81.5 effective individuals (57.5–128.7 95% confidence intervals (CI)) (LD method) and 33.0 (0.0–165.7 (95% CI)) (MC method) (Figure 4). Overall Ne estimates appeared to decline over time across all cohorts (Figure 4) and associated 95% CIs declined congruently. Fluctuation in the CI of the MC method also declined but the upper bound for the 95% CI for the LD method remained infinite across all cohorts (and are not shown in Figure 4). Superficially this appears to indicate a decline but the observed values were not statistically significant.

### 3.3. Population Structure, Migration, and Dispersal

**Figure 5:**
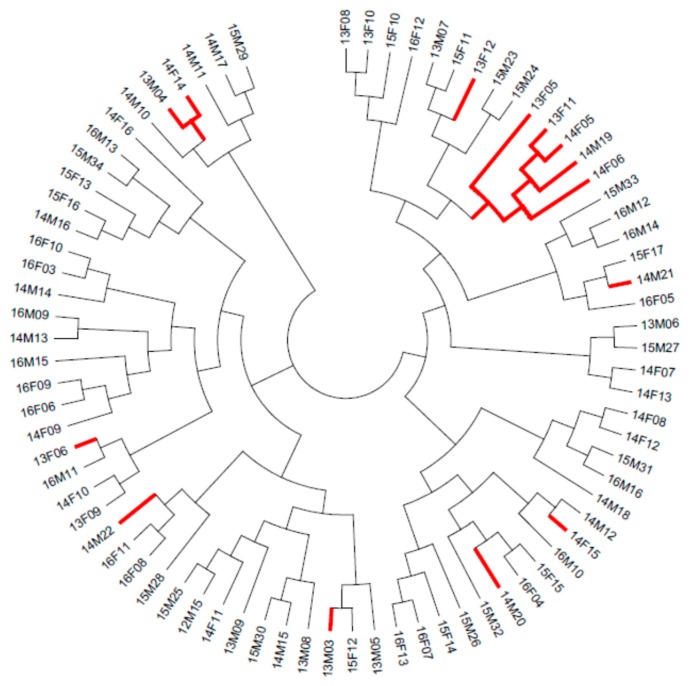
Phylogenetic (Neighbor-Joining) Tree showing all individuals analysed in this study and the relatedness between ‘marsh’ and ‘camps’ populations. Branches highlighted red indicate individuals assigned to ‘camps’ population.

Estimates of migration rate using the private allele method in GENEPOP estimated Nm as 3.28 effective migrants per generation between the ‘marsh’ and ‘camps’ populations. However, the FST method produced a much higher estimate of 20.58. Modelling in STRUCTURE found no evidence for geographic structure across the Marsh area, which was largely supported by the construction of a Neighbor-Joining phylogenetic tree (Figure 5), with individuals from the ‘camps’ population assigned to branches throughout the tree. However, five individuals from the vicinity of Karntama Village (Christmas Creek) were observed to cluster on one branch of the tree, indicating a high degree of relatedness between these individuals.

**Figure 6:**
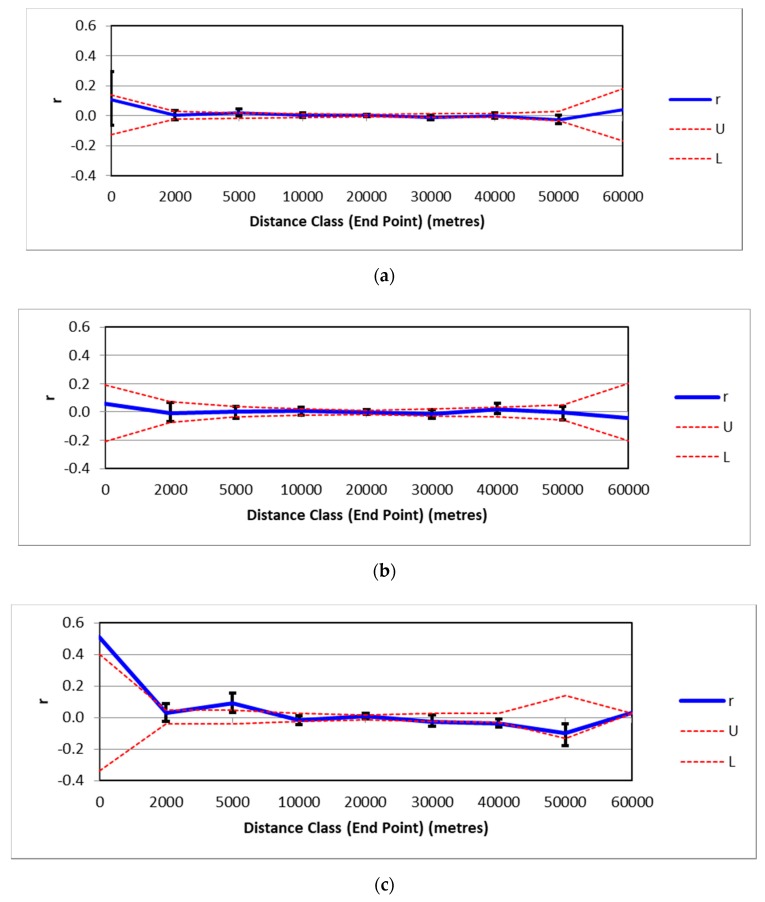
Estimates of spatial autocorrelation between genetic and geographic distances for (**a**) all animals sampled (**b**) males only and (**c**) females only. r = calculated value of relatedness and the x axis is distance classes from 0 to 60,000 m; U and L represent the upper and lower 95% confidence intervals for the null hypothesis of random distribution of cats, outside which values for r can be considered statistically significant at *p* < 0.05 level.

Spatial autocorrelation analysis in GENALEX appeared to support the lack of genetic structure across the landscape, with values for relatedness (r) not fluctuating outside the confidence limits, indicating no correlation between genetic and geographic distance (Figure 6). However, while this result was mirrored when just male cats were analysed (Figure 6), when just females were analysed there was a significant correlation between genetic and geographic distance across distance classes from 0–5 km and 30–40 km (Figure 6).

## 4. Discussion

The Fortescue Marsh area was subject to the broadcast of toxic baits over a five-year period, targeting the feral cat population in the vicinity of the Marsh. The success of annual baiting implementation was monitored using camera traps and occupancy modelling [9,24] and found a significant decrease in post-bait occupancy in each of the five years of the program. However, these methods were not comparable over consecutive years. We investigated whether assessing the changes in genetics of a population that is subject to baiting might provide managers with a tool to monitor the impact of baiting over extended time periods, which may be complementary to other temporal methods used.

Differences in genetic diversity were observed between the putative ‘marsh’ and ‘camps’ populations of feral cats, with the ‘marsh’ population having greater variation across all loci analysed. Generally feral cat populations on the Australian continent exhibit high genetic diversity [47], making the low diversity observed for the ‘camps’ population potentially of significance. However, while the ‘marsh’ sample size is projected to have captured the majority of available diversity in this population, extrapolation for the ‘camps’ population suggests a larger sample is required to achieve this. In spite of this, departures from HWE were highest for the ‘camps’ population, indicating concomitant low heterozygosity and allelic diversity in this population. While inbreeding was predicted for the ‘camps’ population, this was not significant after applying a Bonferroni correction for multiple tests. Observed heterozygosity for the ‘marsh’ population was similar to other studies of feral cat populations (HO = 0.69 cf 0.70 [23,48]) but the ‘camps’ was lower (HO = 0.62).

One key aim of this study was evaluating impacts on genetic diversity between temporal cohorts, but the resulting data were unable to show significant trends that supported any detrimental effect from the baiting program. When estimates were calculated for the three temporal cohorts for the ‘marsh’ population from 2012–2016, allelic diversity appeared to decline with length of time since baiting commencement and FIS also showed an increase over the same period, potentially indicating an increase in inbreeding. However, it was impossible to draw robust conclusions from these results due to the lack of statistical power, which is likely due to a combination of small sample sizes, numbers of markers or the timeframe of the study. Capturing and monitoring feral cats is highly challenging [49] and therefore having the ability to obtain sufficient sample sizes is a critical consideration before embarking on this type of long-term study. Future studies should focus on maximising sample sizes across a larger number (or more temporally discrete) cohorts but this is dependent on the planned duration of the control program. A larger battery of genetic markers for analysis is also recommended, as is consideration for using more sensitive markers such as single-nucleotide polymorphisms (SNPs).

Effective population size is a measure of the contribution of reproductive individuals and is an important population metric. When Ne across all cohorts within the ‘marsh’ population was broken down into three temporal cohorts between 2012 and 2016, Ne estimates appeared to decline with increasing time since baiting commencement in 2012. This, combined with a decline in genetic diversity and/or an increase in FIS, would be indicative of a population that has undergone a rapid contraction or bottleneck [50,51]. However, given the lack of statistical significance, it is impossible to say with confidence that this relationship is not artefactual.

We found no evidence of significant differentiation between the two populations or population substructure. This suggests that the arbitrary allocation of the ‘marsh’ and ‘camps’ populations is not representative of two genetically separate populations. Values for Nm varied widely between models with the private allele method predicting 3.28 effective migrants per generation [37], compared to 20.58 migrants per generation based on FST [38]. Nevertheless, there does appear to be evidence for high gene flow between camp areas and the Marsh as a whole. It is unclear whether areas of mining infrastructure represent an important source of migrants for the rest of the Marsh, but given the apparently high rate of gene flow between the ‘marsh’ and ‘camps’ populations, this would seem to be a likely scenario. A more representative sample of the ‘camps’ population may help clarify this relationship and the removal of feral cats around mining infrastructure could potentially augment the control effort on the Marsh itself.

There was limited evidence of a link between relatedness and geography, with a Neighbor-Joining phylogenetic tree finding little correlation in relatedness between the ‘marsh’ and ‘camps’ populations. However, a group of five individuals from the vicinity of Karntama Village (Christmas Creek) all clustered on the same branch, indicating that populations around mining infrastructure may be related to a higher degree than elsewhere on the Marsh (indeed these individuals were sampled contemporaneously and may be directedly related). This might account for the higher FIS values for the ‘camps’ population, although these results would benefit from a larger sample size for the reasons previously discussed.

Estimates of spatial autocorrelation found no evidence for a relationship between genetic and geographic distance for the overall population and for males as a discrete cohort. However, there was strong evidence for relationship between genetic and geographic distance for female cats. This suggests that female cats in this landscape are at least somewhat philopatric, whereas males appear to disperse more widely, accounting for the absence of any spatial autocorrelation for the male cohort. This is supported by male cats exhibiting, on average, larger home-range sizes than females at Fortescue Marsh over the five years of the program [24]. This difference has also been demonstrated in other studies [52]. Evidence of sex-biased dispersal in feral cats has been reported elsewhere [23] and is suggested to be an inbreeding avoidance mechanism [53]. However, in the context of a long-term baiting program this is important, as differential dispersal rates between sexes may influence how rapid recolonisation of a baited area may occur and how quickly a population can recover from a baiting impact. For example, if female cats are more philopatric than males, areas that are subject to baiting and are recolonised may experience a male biased sex-ratio, which may have an impact on the fertility rate of the population. However, philopatric tendencies may also insulate females somewhat from the impact of baiting, since cats that move less can be assumed to have a lower probability of encountering baits. Of the cats that were collared as part of this program, 50% more male cats died as a result of bait ingestion than females, which supports this hypothesis. Therefore, this may mean that after the recruitment of immigrant males, there is no net change in sex ratio, although more work is required to investigate this more thoroughly. Moreover, if baiting can target periods when females are most active (i.e., least philopatric), (for example, during periods of oestrous) this may help to enhance overall baiting efficacy. Onset of oestrous in cats is thought to be associated with change in daylength in mid-winter in domestic cats [54] and elsewhere in south-eastern Australia [55] and if this was the case in the Pilbara, it would coincide with the lowest period of prey abundance and the current baiting regime. However, further study of this phenomenon would be valuable to confirm if this is the case in northern Australia.

While there is some evidence that canids (e.g., dingo *Canis familiaris dingo*; red fox *Vulpes vulpes*) may influence the behaviour of feral cats [56,57], both species were rarely recorded during the study period, with dingoes being recorded a maximum of three camera locations (out of >68) in any one year and foxes recorded at a maximum of four locations in just two of the five years of the program [24]. Therefore, it is unlikely that canids were responsible for any of the observed impacts on feral cats over the course of the study.

The sampling of genetic material was not the primary reason why cats were captured in this program and this study was ancillary to the main monitoring methods [9,24]. At first glance, the results appear to corroborate with the annual decrease in cat occupancy observed from camera trap data during the same program [9]. Unfortunately, the relatively small sample size and lack of an experimental control mean that these results must be treated with a high degree caution. As discussed, a larger, more long-term study may have yielded more conclusive results. However, we maintain that genetic methods may be a promising solution to providing an empirical measure of the impact of a long-term baiting program, alongside other monitoring strategies such as occupancy modelling and live-trapping [9,24]. Furthermore, if trapping of feral cats is being conducted as part of a control or monitoring program, genetic analysis may represent a cost-effective technique with which to evaluate the program’s long-term success. While the expenditure required for live-trapping, along with the extraction of DNA and subsequent analysis, is not insignificant, the financial investment in the large remote camera array described in [9] (as well as the staff costs to service this array and analyse the images) would certainly be an order of magnitude higher if not more.

## 5. Conclusions

This study demonstrates a long-term monitoring method to evaluate the efficacy of a feral cat control program. However, while our results suggest that genetic analysis could represent a useful monitoring tool, the lack of clear and significant trends highlights the difficulties in using this method for species such as feral cats. A longer-term study, incorporating more generations, larger sample sizes, and larger numbers of more sensitive markers is recommended if attempting to use genetic techniques to monitor impacts of control strategies in this (and other similar) species. The relationship between cats on the Marsh and the mining infrastructure is suggested to be fluid, with high rates of migration per generation which could indicate the mining infrastructure represents a source population that needs to be controlled as part of a broader threat-abatement strategy (e.g., trapping or other removal methods and improved waste management). Finally, we uncover evidence in differential dispersal behaviour between male and female cats, which could have implications for control strategies in this landscape and others.

## Figures and Tables

**Table 1:** Marker names, short-tandem repeat motifs and primer sequences used to genotype 79 individual cats from Fortescue Marsh between 2012 and 2016 [33,34].

Marker	Genbank Accession No.	Repeat Motif	Primer Sequence (5’ to 3’)
FCA126	AF130532	(CA)_24_	F—GCCCCTGATACCCTGAATG
			R—CTATCCTTGCTGGCTGAAGG
F146	AY988112	(GTT)_9_	F—TTACGGTCTCTCCACAAGTC
			R—GAACCAGGTGATGAGAACTG
F164	AY988113	(AAAC)_9_	F—CTATATGACAACTGAGAACT
			R—AGATGATACAGGTAGAGGTC
F27	AY988114	(GAAA)_14_	F—CAGATCACAGTCTTACTGAT
			R—CATTAAATGAGGAAGTACTG
F49	AY988118	(TTG)_8_	F—GTCGAATGCTTAACTGACT
			R—GACATCTGGTCAGTTTCCTC
FCA728	AY988129	(GGAA)_11_	F—TTCAGCTTTTCCTCCTGACAA
			R—CCTGCCTGTATTCCTCACAA
FCA730	AY988131	(GATA)_10_	F—ATTGGGAATTGTAGCCAAGG
			R—CTCCAAGTGGATGGAGCATT
FCA735	AY988136	(CCAT)_6_/(AC)_15_	F—TCAAGGCCAATTGTAGAGCA
			R—TTCCATTCTCTATGGAATAGTCAGT
FCA744	AY988145	(GATA)_9_	F—CATTGGGCCTACAGCCTACT
			R—TCAACACCCTCACACCAATG
FCA747	AY988147	(GATA)_10_	F—GCCTCTTTGGCAACCATTAG
			R—TCTTGGAATTACTCCTGGTAAACA
FCA1059	AY988153	(GAAA)_9_	F—TGAAAAGCATATGCAAAAGTTGA
			R—TCTCCAAATTCCTATCTCACAAC

**Table 2:** Summary of estimates of allelic diversity, observed and expected heterozygosities and F-statistics [46] for the Fortescue Marsh and Fortescue Camps feral cat populations.

Locus	Fortescue Marsh (n = 65)					Fortescue Camps (n = 14)				
	HE	HO	FIS	AL	AR	H_E_	H_O_	FIS	AL/AR	FST
FCA735	42.43	44	−0.037 ^2^	4	3.96	9.04	11	−0.228	3	−0.009
FCA728	38.79	37	0.047	6	4.60	7.96	10	−0.268	3	−0.014
FCA730	52.29	53	0.014	9	6.64	9.33	8	0.148	5	0.049
FCA126	48.94	49	−0.001	7	5.53	9.78	11	−0.130	6	0.055
FCA1059	48.69	42	0.138	6	5.51	11.22	11	0.021	5	−0.004
F146	44.40	42	0.055	4	3.78	10.19	10	0.019	4	−0.006
F27	53.77	48	0.108	11	7.90	11.26	7	0.387	7	0.012
FCA744	41.17	44	−0.069	5	4.49	7.59	3	0.614 ^1^	3	−0.008
F164	49.24	51	−0.036	6	5.57	11.22	11	0.021	6	0.015
FCA747	50.42	44	0.128	7	5.81	10.00	11	−0.104	6	0.016
F49	35.63	40	−0.124	3	3.00	5.89	3	0.500	3	0.007
Mean	45.98	44.91	0.018	6.18	5.16	9.41	8.73	0.036 ^2^	4.64	0.012

HE, expected heterozygosity; HO, observed heterozygosity; FIS, inbreeding coefficient; AL, alleles per locus; AR, allelic richness (as calculated by rarefaction in HP-RARE [35] (rarefaction performed with maximum sample size for Fortescue Camps (n = 14 or 28 genes); FST, pairwise fixation index; ^1^ significant (*p* < 0.05) after Bonferroni correction; ^2^ significant (*p* < 0.05) before Bonferroni correction only.
